# Diagnostic Accuracy of Cough Frequency Monitors: A Systematic Review and Meta‐Analysis

**DOI:** 10.1002/resp.70229

**Published:** 2026-03-08

**Authors:** Ana Lara Castro Rodrigues, Larisse Sousa Reis Passafaro, Daniele Oliveira dos Santos, Marcos Gontijo da Silva, Noé Mitterhofer Eiterer Ponce de Leon da Costa, Janne Marques Silveira, Arietta Spinou, Ada Clarice Gastaldi

**Affiliations:** ^1^ Graduate Program in Rehabilitation and Functional Performance, Ribeirão Preto Medical School (FMRP‐USP) University of São Paulo São Paulo Brazil; ^2^ Hospital das Clinicas of the Medicine School in Ribeirão Preto University of São Paulo São Paulo Brazil; ^3^ Graduate Program in Biotechnology (PPGBIOTEC) Federal University of Tocantins (UFT) Tocantins Brazil; ^4^ Applied Statistics and Biometrics. Graduate Program in Biotechnology (PPGBIOTEC) Federal University of Tocantins (UFT) Tocantins Brazil; ^5^ Department of Health—Medicine and Physiotherapy University of Gurupi Tocantins Brazil; ^6^ Respiratory Physiotherapy School of Life Course & Population Sciences Faculty of Life Sciences & Medicine, King's College London London UK

**Keywords:** cough, cough assessment, cough counting, cough frequency, cough monitor, diagnostic accuracy, monitoring tools

## Abstract

**Background and Objective:**

International guidelines emphasise the importance of objective cough assessment for evaluating therapeutic efficacy. Cough negatively impacts quality of life and generates high costs for healthcare systems. However, the accuracy of cough frequency monitors remains unclear. This study aimed to identify tools developed to monitor cough frequency and evaluate their accuracy.

**Methods:**

Observational studies involving adults, children, or infants who used cough frequency monitors were included from the Pubmed/MEDLINE, EMBASE, and Web of Science databases published up to 24 November 2024. Two independent reviewers performed the screening, data extraction, and quality assessment of the studies using QUADAS‐2. Summary estimates of diagnostic accuracy were calculated with meta‐analysis using bivariate mixed effects regression.

**Results:**

Nineteen studies were included in the review. In the 16 studies included in the meta‐analysis with 52,612 events, sensitivity was 89% (95% CI 0.84–0.93, *I* ^2^ 89.3%) and specificity was 99% (95% CI 0.98–1.00, *I* ^2^ 88.2%). The area under the curve (AUC) of 0.96 indicated excellent discriminative capacity. In the 11 devices and 4 applications identified, individual accuracy was > 0.9.

**Conclusion:**

The tools are accurate in distinguishing between cough and non‐cough sounds, but their clinical application still presents technical challenges and requires investment. Future research should improve the monitors, maintaining high accuracy but allowing for agile, cost‐effective assessment.

## 1 Introduction

Cough is a reflex, essential for clearing of the airway secretions, and the defence and maintenance of pulmonary homeostasis under physiological conditions [1]. Cough is a symptom present in a variety of pulmonary and extrapulmonary diseases [2]. Its prevalence is estimated at 2%–18% in adults and up to 30% in children [3, 4].

As a symptom it compromises patients' quality of life (QoL), social interactions and well‐being [5]. It also has a significant impact on healthcare costs due to required tests, medication, and health consultations [6]. Despite this, its effects on health status are rarely measured in clinical practice [7].

Cough can be assessed using subjective scores, quality of life (QoL) questionnaires, whilst objective assessment incorporates monitors or manual counting, which is considered the gold standard [8]. Studies show a low correlation between subjective and objective measurement methods [9]. Cough monitor measures cough frequency, its impact on QoL to be assessed, and the response to treatment to be evaluated [8]. Technology is quickly adopted within the healthcare and telemonitoring tools are already used since they offer established advantages, such as optimization of resources and time allocation and better continuity of treatment [10].

Despite technological advances and international guidelines recommending objective cough monitoring to assess treatment effectiveness in respiratory diseases [2], the use of cough frequency monitors remains limited in clinical practice [7]. Although previous reviews have explored different cough monitoring technologies [7, 8, 11], we found no systematic review reporting a diagnostic accuracy meta‐analysis directly comparing these devices with the gold standard. Part of this limited use may be explained by the lack of evidence on whether these monitors provide accuracy comparable to manual counting, which reinforces the importance of synthesising available studies to promote clinical implementation and support decision‐making.

Therefore, the aim of this systematic review and meta‐analysis is to identify the available tools for monitoring cough frequency and evaluate their accuracy, sensitivity, and specificity in detecting cough.

## 2 Methods

The systematic review followed the PRISMA DTA guidelines [12] and was registered in the International Prospective Registry of Systematic Reviews (PROSPERO) with registration number CRD42020187919.

### 2.1 Eligibility Criteria

Cross‐sectional observational studies of the diagnostic accuracy of devices or mobile applications that perform automatic or semi‐automatic cough frequency counting were included, using manual counting as the reference standard [8, 10], involving adults, children, and infants, with or without cough, in hospital, outpatient, or community settings. The primary outcomes were sensitivity and specificity. Exclusion criteria were conference abstracts, study protocols, grey literature, and studies not available in English.

### 2.2 Information Sources and Search

A search strategy was developed for each database, PubMed/MEDLINE, Web of Science, and EMBASE using MeSH terms. The last date of literature search was 24 November 2024, and keywords included: ‘cough’ AND ‘monitoring’ AND ‘sensitivity’ OR ‘specificity’. No filters were applied, as recommended for systematic reviews of diagnostic accuracy [13]. The full search strategies are provided in the Supporting Information S1 and S2. Additionally, the reference lists of the included records were hand searched to identify further eligible studies [14].

### 2.3 Data Selection and Extraction

Rayyan software was used and duplicated studies were deleted [15]. Screening, study selection, and data extraction were performed by independent reviewers (ALCR, JMS, LSRP), and discrepancies were resolved by a third reviewer (DOS). The data collected were: author, year of publication, country of study, study design, characteristics of the monitor, validation participants, cough monitor variables (name, components and cough identification automation, cough monitor positioning), cough frequency, monitoring capture (total time, time of the day, monitoring location), comparator used for validation, sensitivity, specificity, true positive (TP), false negative (FN), true negative (TN), and false positive (FP) data. The study authors were contacted when data was not available in the publications.

### 2.4 Risk of Bias Assessment

The risk of bias for individual studies was assessed using Quality Assessment of Diagnostic Accuracy Studies 2 (QUADAS‐2) [16] and classified as high, low, or uncertain in four domains (patient selection, index test, reference test, and flow and time). Concerns about applicability were classified in the first three domains. The assessment was conducted by independent reviewers, with discrepancies resolved by a third reviewer. The risk of bias graphs were prepared using Microsoft Excel 2019 (Microsoft Corporation).

### 2.5 Appraisal of the Certainty of Evidence

The certainty of the evidence was assessed using the Grading of Recommendations, Assessment, Development, and Evaluation (GRADE) [17].

### 2.6 Summary Measures and Synthesis of Results

Data analysis was performed using R statistical language (version 4.5.2; R Core Team) within the RStudio environment (2025.09.2; Posit Team). Diagnostic accuracy was synthesised using the bivariate random‐effects model of Reitsma to jointly pool sensitivity and specificity across studies [18]. The model was implemented with the reitsma function from the mada package (version 2024.0.5.12), with parameters estimated by restricted maximum likelihood (REML). Direct URLs for all software and packages cited are provided in the Supporting Information S3. The summary receiver operating characteristic (SROC) curve was derived using the sroc function based on the approach of Rutther and Gatsonis [19]. Diagnostic accuracy for each individual study was calculated using the standard formula (TP + TN)/(TP + TN + FP + FN) [20]. All accuracy metrics were derived programmatically in R to ensure reproducibility. The model calculated positive (LR+) and negative (LR‐) likelihood ratios, the diagnostic odds ratio (DOR) (scoring values in the Supporting Information S4), and diagnostic accuracy.

Meta‐analyses were conducted for data reported in more than three studies. Sensitivity and specificity outcomes were expressed in 95% confidence intervals (95% CI) for the meta‐analysis. Between‐study heterogeneity was assessed within a frequentist framework [18]. As an initial description of variability, heterogeneity in sensitivity and specificity was quantified separately using *I* ^2^ statistics, with interpretation based on Higgins and Thompson [21]. To better characterise heterogeneity within the bivariate diagnostic accuracy model, the between‐study variance components (*τ* ^2^) for sensitivity and specificity on the logit scale were estimated directly from Reitsma's random‐effects model and reported. For *I* ^2^, values < 10% were considered to indicate low heterogeneity and values > 10% the presence of heterogeneity, in conjunction with the Cochran *Q* test.

Sensitivity and subgroup analyses were conducted to assess the stability of the pooled estimates across relevant study characteristics: (1) participant health status, categorised as patients, healthy individuals or mixed group. Patients were defined as individuals with clinical conditions that may affect cough characteristics, including respiratory diseases, neurological disorders, or acute clinical conditions. Healthy individuals had no known conditions affecting cough. Mixed groups included studies that evaluated patients and healthy individuals within the same cohort; (2) cough collection environment, categorised as clinical or home; (3) monitor position, categorised as contact with the participant's skin or in the environment; (4) type of cough frequency identification, categorised as automatic or semi‐automatic; (5) type of cough, categorised as spontaneous or volitional.

Forest plots were used to visually represent the distributions of sensitivity and specificity among studies and their pooled estimates, as well as heterogeneity. Overall test performance was assessed using the summary receiver operating characteristic (SROC) curve derived from the bivariate random‐effects model [18, 19]. The discriminative capacity, represented by the area under the curve (AUC) was classified as low (AUC < 0.7), moderate (0.7 ≤ AUC < 0.9) or high (AUC ≥ 0.9) [22].

To assess potential small‐study effects, Deeks' funnel plot asymmetry test was applied [23]. Asymmetry was further explored through influence analyses based on studentized residuals from the random‐effects model, with absolute z‐scores > 2.0 indicating potentially influential studies. The impact of these studies on the overall model and funnel plot asymmetry was evaluated using influence diagnostics [24, 25] and sensitivity analyses were conducted to assess the robustness of the pooled estimates [26].

## 3 Results

### 3.1 Study Selection

**FIGURE 1:**
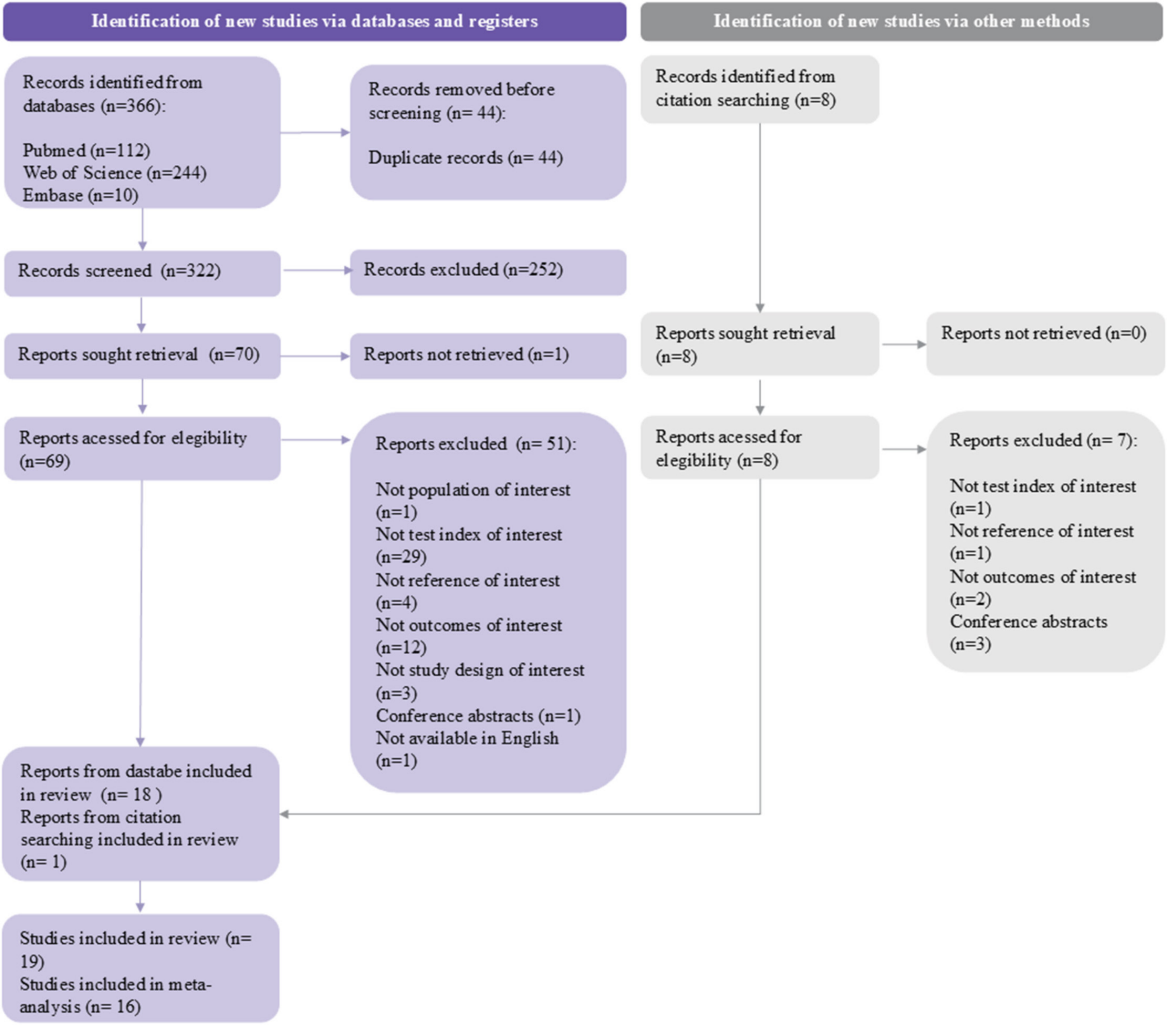
PRISMA flowchart.

The literature search identified 366 publications. After excluding 44 duplicates and screening titles and abstracts, 70 articles were considered eligible for full‐text screening. Eighteen articles were included. The manual search of references resulted in eight studies, of which 1 study was included after full‐text reading (Figure 1). Of the 19 studies included in the systematic review (Table 1), 16 were included in the meta‐analyses, with 413 participants and 52,612 events.

**TABLE 1:** Study characteristics.

Author, year	Country	Monitor	n*	Patient health	Collection location	Device position	Type of equipment	Type of cough	Accuracy %
Barata et al.; 2023 a	Switzerland	Computer	9	Pneumonia	Clinical environment	Positioning in the environment	Automatic	Spontaneous	93.6
Barata et al.; 2023 b	Switzerland	Smartphone in real time	9	Pneumonia	Clinical environment	Positioning in the environment	Automatic	Spontaneous	92.3
Barry et al.; 2006	UK	HACC	10	Smokers	Clinical environment	Positioned on the skin	Semi‐automatic	Spontaneous	92.8
Birring et al.; 2008 a	UK	LCM	9	Chronic cough	Clinical environment	Positioned on the skin	Semi‐automatic	Spontaneous	97.8
Birring et al.; 2008 b	UK	LCM	8	Healthy	Clinical environment	Positioned on the skin	Semi‐automatic	Spontaneous	97.7
Coyle et al.; 2005	USA	LifeShirt	8	COPD	Clinical environment	Positioned on the skin	Automatic	Spontaneous	97.4
Corrigan and Paton; 2003	USA	LR100	26	Healthy infants and infants with cough	Clinical environment	Positioned on the skin	Automatic	Spontaneous	—
Cuong; 2016	Vietnam	MobiCough	10	Pharyngitis and pneumonia	Not reported	Positioned on the skin	Automatic	Spontaneous	—
Do et al.; 2022	UK	Albuns Home RD	10	Respiratory diseases	Home	Positioning in the environment	Semi‐automatic	Spontaneous	99.5
Hoyos‐Barceló et al.; 2018	UK	Smart APP	13	COPD, bronchiectasis and asthma	Clinical environment	Positioning in the environment	Automatic	Volitional	100
Kadambi et al.; 2010	UK	VitaloJAK + algorithm	9	Chronic cough	Home	Positioned on the skin	Automatic	Spontaneous	97.2
Krajnik et al.; 2010	Poland	MES Cough Analyser.	13	Healthy	Home	Positioned on the skin	Automatic	Spontaneous	99.7
Kuhn et al.; 2023 a	Switzerland	SIVA‐P3 Day	27	Chronic cough	Home	Positioned on the skin	Automatic	Spontaneous	100
Kuhn et al.; 2023 b	Switzerland	SIVA‐P3 Night	27	Chronic cough	Home	Positioned on the skin	Automatic	Spontaneous	96.7
Kulnik et al.; 2016	UK	LCM	5	Hospitalised post‐stroke	Clinical environment	Positioned on the skin	Automatic	Volitional	91.0
Larson et al.; 2012	Peru	CayeCoM	15	Tuberculosis	Clinical environment	Positioned on the skin	Semi‐automatic	Spontaneous	97.0
Matos et al.; 2007	UK	LCM	26	Healthy and respiratory diseases	Clinical environment	Positioned on the skin	Semi‐automatic	Spontaneous	98.4
Otoshi et al.; 2021	Japan	—	21	Healthy and chronic cough	Clinical environment	Positioned on the skin	Automatic	Volitional	96.1
Stevens et al.; 2024	Belgium	MoveSense MD	38	Healthy	Clinical environment	Positioned on the skin	Automatic	Volitional	91.4
Urban et al.; 2022	Germany	LEOSound	98	Healthy children and children with respiratory diseases	Home	Positioned on the skin	Automatic	Spontaneous	98.7
Turner et al.; 2014	UK	PulmoTrack‐CC	10	Chronic cough	Clinical environment	Positioned on the skin	Automatic	Spontaneous	—
Vizel et al.; 2010	Israel	PulmoTrack‐CC	12	Healthy	Clinical environment	Positioned on the skin	Automatic	Volitional	100

Abbreviations: COPD: Chronic obstructive pulmonary disease; n*: number of individuals; UK: United Kingdom; USA: United States of America; —: studies that were not included in the meta‐analysis.

### 3.2 Study Characteristics

Sixteen included studies presented both sensitivity and specificity data and were also included in the meta‐analysis [27, 28, 29, 30, 31, 32, 33, 34, 35, 36, 37, 38, 39, 40, 41, 42]. The characteristics of the included studies are shown in Table 1. Of the 16 of the meta‐analysis, three presented sensitivity and specificity outcomes in different contexts. Barata et al. evaluated the accuracy of the software on mobile phones and computers [31]; Birring et al. evaluated the accuracy of the device in healthy individuals and patients with chronic cough [33]; and Kuhn et al. evaluated the accuracy of the device during the day and at night [29]. The results of these outcomes were evaluated separately in the meta‐analysis (a and b). Three studies were included in the systematic review but presented only sensitivity data [43, 44, 45].

Cross‐sectional observational studies were included, and all 19 used the gold standard for monitor validation, manual counting [27, 28, 29, 30, 31, 32, 33, 34, 35, 36, 37, 38, 39, 40, 41, 42, 43, 44, 45]. Eleven devices for objective cough assessment were identified [27, 28, 29, 30, 32, 33, 34, 36, 37, 38, 39, 41, 42], including four mobile or computer applications [31, 35, 40, 44]. A total of 413 participants were included, including both healthy individuals and patients [27, 28, 29, 30, 31, 32, 33, 34, 35, 36, 37, 38, 39, 40, 41, 42, 43, 44, 45]. Regarding the cough collection environment, 13 studies monitored coughing in a clinical setting [30, 31, 32, 33, 34, 35, 37, 38, 39, 40, 42, 43, 45], five studies monitored coughing in the home environment [27, 28, 29, 36, 41] and only one did not report the monitoring location [44]. In 16 studies, the device was placed directly on the patient's skin [28, 29, 30, 32, 33, 34, 36, 37, 38, 39, 40, 41, 42, 43, 44, 45], while in 3 it was placed in the environment close to the patient [27, 31, 35]. In 14 studies, monitoring was fully automatic [28, 29, 31, 34, 35, 36, 37, 39, 40, 41, 42, 43, 44, 45] and in five other studies, monitoring was semi‐automatic [27, 30, 32, 33, 38]. Five studies validated the monitors with volitional coughing [35, 37, 39, 40, 42], while 14 used spontaneous coughing for validation [27, 28, 29, 30, 31, 32, 33, 34, 36, 38, 41, 43, 44, 45].

### 3.3 Risk of Bias Assessment

**FIGURE 2:**
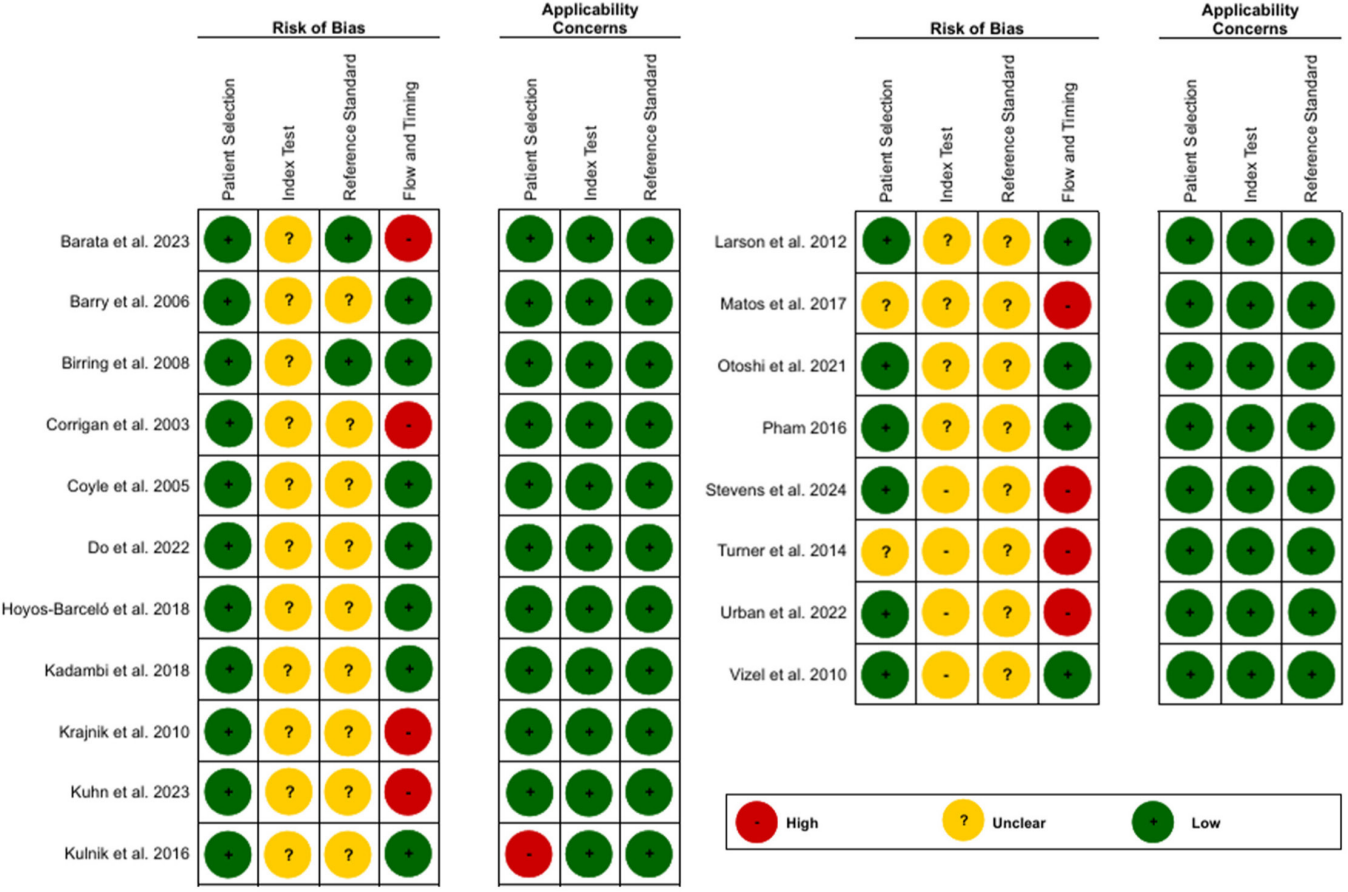
Risk of bias using the Quality Assessment of Diagnostic Accuracy Studies‐2 (QUADAS‐2).

All studies that were included in the systematic review were assessed for risk of bias [27, 28, 29, 30, 31, 32, 33, 34, 35, 36, 37, 38, 39, 40, 41, 42, 43, 44, 45] (Figure 2 and Supporting Information S6) In the domain of patient selection, 17 studies had low risk [27, 28, 29, 31, 32, 33, 34, 35, 36, 37, 38, 39, 40, 41, 42, 43, 44] and two studies presented uncertain risk of bias [30, 45]. For the index test, the risk of bias was categorised as uncertain in all studies [27, 28, 29, 30, 31, 32, 33, 34, 35, 36, 37, 38, 39, 40, 41, 42, 43, 44, 45]. In the reference test domain, only two studies presented low risk [32, 33], while the remaining 17 studies had uncertain risk of bias [27, 28, 29, 30, 31, 34, 35, 36, 37, 38, 39, 40, 41, 42, 43, 44, 45]. Regarding flow and time, 11 studies had low risk [27, 32, 33, 34, 35, 36, 37, 38, 39, 42, 44], and eight studies presented high risk of bias [28, 31, 40, 41, 43, 45]. In the assessment of applicability, only one study presented high concern, identified in the applicability in the patient selection [37], while the remaining studies presented low concern for applicability [27, 28, 29, 30, 31, 32, 33, 34, 35, 36, 38, 39, 40, 41, 42, 43, 44, 45]. With regard to the test index and test reference domains, all studies showed low concern with applicability in both domains [27, 28, 29, 30, 31, 32, 33, 34, 35, 36, 37, 38, 39, 40, 41, 42, 43, 44, 45].

### 3.4 Sensitivity, Specificity, and Accuracy

**FIGURE 3:**
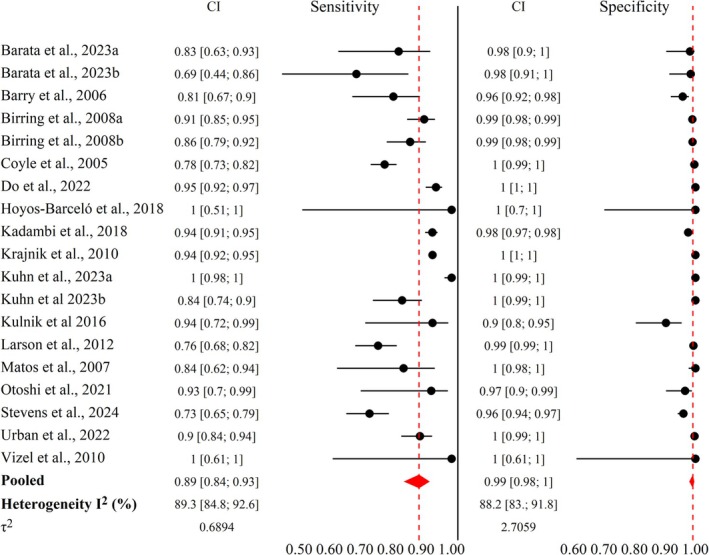
Meta‐analysis of sensitivity and specificity of cough monitoring systems (*n* = 16 studies).

In the meta‐analysis, 16 studies were included [27, 28, 29, 30, 31, 32, 33, 34, 35, 36, 37, 38, 39, 40, 41, 42] reporting 52,612 events, the sensitivity of the monitors was 89% (*I* ^2^ 89%) and specificity was 99% (*I* ^2^ 88.2%) (Figure 3), with moderate certainty of evidence for both outcomes (Supporting Information S17). Raw data extracted from the articles included in 2 × 2 tables to assess diagnostic accuracy are in the Supporting Information S7.

**FIGURE 4:**
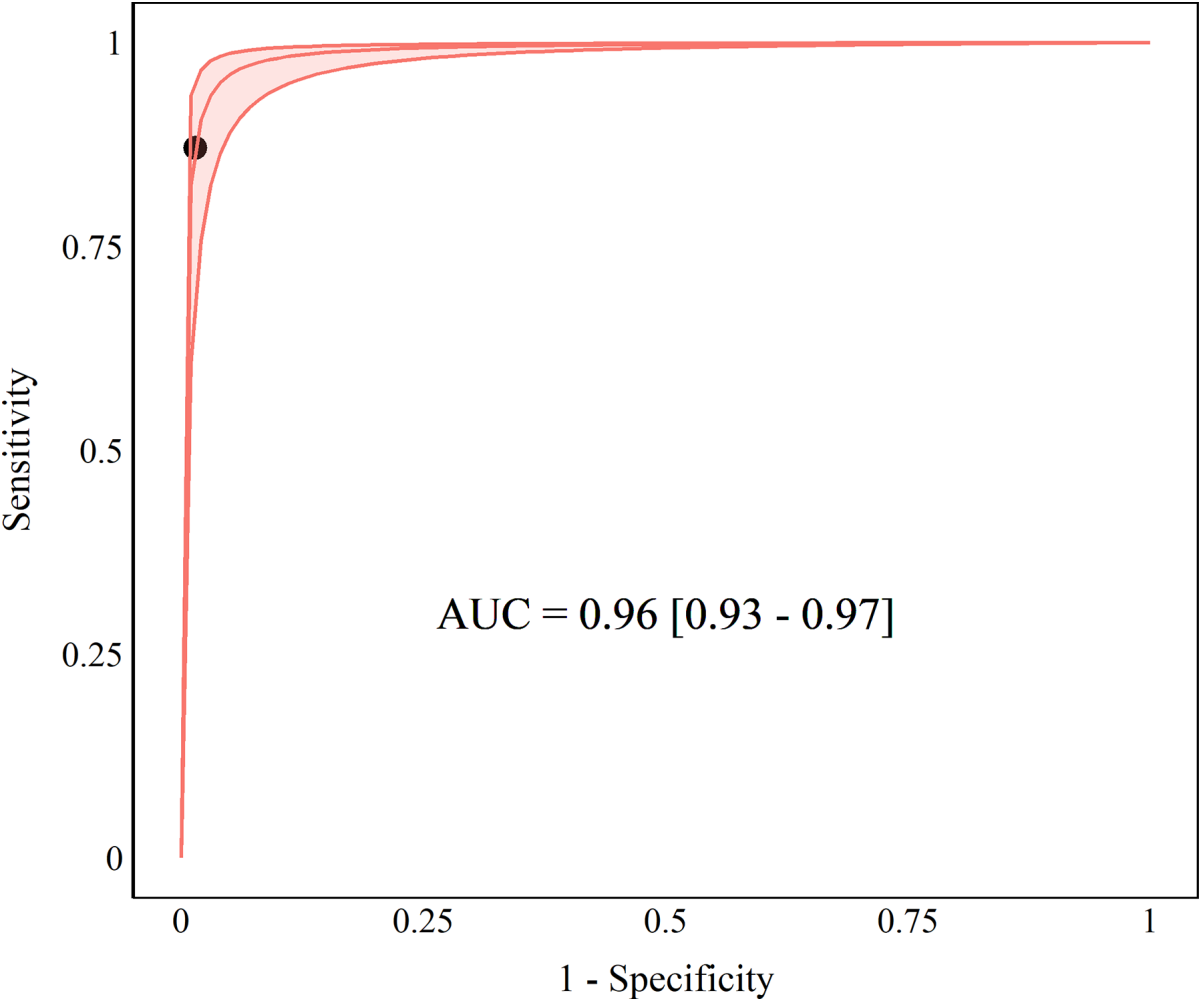
Summary receiver operating characteristic (SROC) curve of sensitivity and specificity.

The positive likelihood ratio (LR+) was 41.22 (95% CI 35.00–83.56), and the negative likelihood ratio (LR‐) was 0.09 (95% CI 0.05–0.15), both derived from sensitivity and specificity. The pooled diagnostic odds ratio (DOR), derived from the likelihood ratios, was 977.34 (95% CI: 333.95–2860.35). The discriminative capacity was classified as high (AUC 0.96, 95% CI 0.93–0.97) (Figure 4). Based on pooled likelihood ratios and an assumed average prevalence of 7.43%, the Positive Predictive Value (PPV) was 87.71% and the Negative Predictive Value (NPV) was 99.12% (Supporting Information S17). The individual accuracy of the studies ranged from 0.92 to 1 (Table 1).

### 3.5 Sensitivity Analysis

Subgroup sensitivity analyses (Table 2) demonstrated a reduction in heterogeneity for sensitivity outcomes. Heterogeneity decreased from 89.3% (Figure 3) to 0% in the health status subgroup in studies that included both healthy individuals and patients, defined as participants with respiratory, neurological, or acute clinical conditions that may influence cough characteristics (Supporting Information S8). Reduced heterogeneity to 19.3% was also observed in the volitional cough type subgroup, in which cough was elicited by the researcher rather than occurring spontaneously (Supporting Information S12). Reductions in heterogeneity were also observed according to the assessment setting, decreasing to 35.3% in clinical environments and to 67.4% in home settings (Supporting Information S9).

**TABLE 2:** Overall and subgroup analyses for diagnostic accuracy.

General conditions and subgroups	n*	Combined sensitivity			Combined specificity			Positive likelihood ratio (LR+)	Negative likelihood ratio (LR‐)	Combined DOR			AUC
		Value (95% CI)	*I* ^2^ (%)	*τ* ^2^	Value (95% CI)	*I* ^2^ (%)	*τ* ^2^	Value (95% CI)	Value (95% CI)	Value (95% CI)	*I* ^2^ (%)	*τ* ^2^	
Participants health status													
Patients	12	0.89 (0.81–0.94)	85.7	0.87	0.99 (0.97–1.0)	88.2	1.53	76.89 (66.41–89.04)	0.12 (0.10–0.13)	698.28 (275.71–1768.49)	72.2	2.04	0.977
Healthy individuals	4	0.88 (0.77–0.94)	95.7	0.73	0.99 (0.84–1.0)	92.5	19.85	1149.0 (769.75–1715.34)	0.08 (0.07–0.09)	2090.66 (5.29–825883.36)	95.4	25.89	0.995
Mixed group	3	0.89 (0.84–0.93)	0	0.89	0.99 (0.96–1.0)	81.1	1.76	11.45 (54.65–227.35)	0.11 (0.07–0.17)	1625.45 (467.32–5653.65)	10.0	0.33	0.985
Collection location													
Clinical environment	13	0.81 (0.77–0.85)	35.3	0.07	0.98 (0.97–0.99)	86.3	0.94	72.46 (58.65–89.57)	0.20 (0.17–0.22)	299.78 (157.79–569.56)	79.1	0.75	0.967
Home	6	0.94 (0.88–0.97)	67.3	0.78	1 (0.99–1.0)	92.0	5.34	287.15 (241.56–341.50)	0.06 (0.06–0.07)	17,725.95 (1539.04–201459.67)	90.4	7.75	0.993
Device position													
Positioned on the skin	15	0.89 (0.83–0.93)	90.6	0.70	0.99 (0.98–1.0)	90.1	2.83	190.7 (166.7–6218.66)	0.10 (0.09–10.11)	967.19 (299.89–3119.37)	88.3	4.39	0.987
Positioning in the environment	4	0.89 (0.74–0.95)	79.4	0.60	0.99 (0.93–1.0)	68.3	3.66	790.00 (255.07–2451.12)	0.08 (0.05–0.11)	854.21 (40.21–18,144.26)	79.3	7.61	0.991
Type of equipment													
Automatic	13	0.91 (0.83–0.95)	91.0	1.07	0.99 (0.97–1.0)	89.5	3.71	211.46 (182.49–245.09)	0.09 (0.08–0.10)	1006.78 (227.73–4450.83)	89.2	6.01	0.989
Semi‐automatic	6	0.87 (0.80–0.92)	84.6	0.31	0.99 (0.98–1.0)	80.4	1.32	141.58 (102.59–195.49)	0.12 (0.10–0.15)	911.38 (196.87–4219.15)	80.8	3.00	0.981
Type of cough													
Spontaneous	14	0.89 (0.84–0.93)	90.4	0.69	1 (0.99–1.0)	88.9	2.41	205.62 (177.63–238.07)	0.09 (0.08–0.10)	1797.68 (486.57–6641.70)	82.6	5.24	0.989
Volitional	5	0.90 (0.68–0.98)	19.3	0.78	0.95 (0.91–0.97)	9.3	0.13	17.63 (12.58–24.69)	0.24 (0.18–0.31)	96.29 (41.38–224.05)	0.0	0.20	0.912
Total patients (overall)	19	0.89 (0.84–0.93)	89.3	0.69	0.99 (0.98–1.0)	88.2	2.71	41.22 (35–88.36)	0.09 (0.05–0.15)	977.34 (333.95–4860.35)	86.9	4.53	0.966

Abbreviations: AUC: area under the curve; DOR: diagnostic odds ratio; n* number of studies with outcome.

Sensitivity analyses for the specificity by subgroups also demonstrated reduced heterogeneity. Heterogeneity decreased from 88.2% (Figure 3) to 68.3% in the subgroup defined by device position on the patient's skin, in which the cough frequency monitor was attached directly to the participant rather than placed in the surrounding environment (Supporting Information S10). A further reduction in heterogeneity was observed to 9.3% in the volitional cough type subgroup (Supporting Information S12). All subgroup sensitivity results are presented in Table 2.

### 3.6 Publication Bias

Deeks' asymmetry test indicated funnel plot asymmetry in the primary analysis (*p* = 0.0153; Supporting Information S13). Influence diagnostics based on studentized residuals identified the study by Krajnik et al. as highly influential (*z* = 3.59) (Supporting Information S14); notably, this study did not receive industry funding. After exclusion of this study, Deeks' test no longer suggested statistically significant asymmetry (*p* = 0.6788; Supporting Information S15).

Sensitivity analysis excluding Krajnik et al. [28] showed a pooled sensitivity of 0.89 (95% CI: 0.83–0.93; *I* ^2^ = 83.1%; *τ* ^2^ = 0.71) and a pooled specificity of 0.99 (95% CI: 0.98–0.99; *I* ^2^ = 86.6%; *τ* ^2^ = 1.40), with estimates similar to those of the primary meta‐analysis (Supporting Information S16).

## 4 Discussion

This systematic review identified 11 devices [27, 28, 29, 30, 32, 33, 34, 36, 37, 38, 39, 41, 42], three mobile applications [35, 40, 44] and one application that can be installed on a mobile phone or computer [31] for the objective assessment of cough frequency. The meta‐analysis demonstrated excellent accuracy of the cough frequency monitors. The individual accuracy of the studies did not demonstrate inferiority in any of the devices evaluated.

The certainty of the evidence, using GRADE, was moderate. This rating was mainly due to the observational design of the studies, the high risk of bias caused by the absence of clear cut‐off points, and the heterogeneity across studies. This heterogeneity was largely explained by the lack of standardisation regarding clinical differences. The studies included healthy individuals and patients, different intervention settings (clinical or home environment), as well as different types of cough (voluntary or spontaneous).

Still, sensitivity and specificity were consistent in the subgroup that evaluated only the volitional cough. This finding suggests that the main source of heterogeneity is the type of cough analysed, and not in the cough frequency monitor used. This is consistent with guidelines from other authors [46] that algorithms should be customised to adapt to the cough sounds of the subject under observation.

In this review, the Leicester Cough Monitor (LCM) [30, 33, 36] was the only cough frequency monitor that presented validation studies in different contexts and populations. In addition, human input during calibration of the automatic identification process allows the monitor to classify a small fraction of the detected sounds as coughs or not using a verifier. This input is used to create tailored statistical models. These models are adapted to the audio features of the cough sounds for each specific recording and therefore individual.

Although diagnostic accuracy studies define ‘a priori’ the ideal cut‐off point for optimal sensitivity and specificity, this is rarely in cough frequency monitor validation studies. The intensity of the cough sound correlates strongly with the peak cough flow [47]. Regarding coughing, it is important to note that peak cough flow can vary from one patient to another, depending on factors such as gender, age, and height [48]. In healthy individuals, volitional cough may generate higher peak values, especially when compared to those of patients with neuromuscular diseases [48]. This difference may justify the slightly higher AUC in the subgroup of healthy individuals.

Customization of cough frequency monitors according to the health status of the individual was identified by Hall et al. [46]. This approach may be useful in the development of devices or applications that, in addition to monitoring coughing, aim to also diagnose diseases causing cough or act as a marker of severity [46]. The potential adaptation of mobile devices through validated applications for cough monitoring is therefore very attractive [46]. However, cough processing stages must be considered. Different recording methods can influence the sensitivity of the monitor when the device was evaluated separately [31, 35, 40, 44].

To our knowledge, no cough frequency monitor has been approved by a regulatory agency for clinical use. The primary validation study of VitaloJAK [49] reported a sensitivity ranging from 99.2% to 100%. However, this study was excluded from this review because it was a conference abstract without complete data for analysis. The validation of its automatic version was included [36]. Some monitors, such as VitaloJAK, are approved for sound recording, but not as validated cough frequency monitors. This approval does not guarantee the accuracy of the software for drug evaluation or for decision‐making in clinical practice.

There are no reports on the cost of developing the devices included in this review. Hall et al. point out that, despite technological advances in the last two decades, there has been little financial investment in cough frequency monitors [46]. This may explain the difficulty in finding devices with robust validation being used in the evaluation of new therapeutic interventions for cough. Examples of monitors with interrupted development include the LR100 [43], one of the first monitors developed, and the LifeShirt [34], which was discontinued after the company closed. These examples highlight the importance of investing resources from diverse sources.

One of the strengths of this systematic review is its rigorous methodology and the inclusion of only studies that used the gold standard of manual counting for validating their cough frequency monitors. In line with this, the QUADAS II showed low concern regarding the applicability of the studies. Beyond sensitivity and specificity, test performance was summarised using likelihood ratios, the diagnostic odds ratio, and the area under the summary ROC (SROC) curve. While AUC reflects discriminative capacity, clinical performance was primarily interpreted using threshold‐specific sensitivity and specificity.

Our review focused on peer‐reviewed evidence, with well‐defined eligibility and analysis criteria. Therefore, we did not include grey literature, which may have excluded some ongoing or locally available systems. Although the sources of financial investment in the development of the monitor were addressed and bias risk analysis was performed, it was not possible to conduct an ad hoc economic analysis of the included studies due to insufficient data availability. Furthermore, no evidence of relevant small‐study effects was identified by funnel plot asymmetry, although the interpretation of such analyses in diagnostic test accuracy meta‐analyses should be made with caution [26].

Most validation studies did not present sample size calculations and included a small number of participants. There is potential instability in heterogeneity estimates in subgroups with a limited number of studies. In addition, the methods used to calculate accuracy were not clearly defined, constituting important limitations. As a result, the high accuracy reported in controlled validation settings may not fully reflect real‐world performance, where methodological and statistical variability is greater. Furthermore, the methodological quality and small sample sizes of the included studies compromised the certainty of the evidence. Future studies should clearly define and transparently report methods for calculating accuracy and subsequently focus on the economic feasibility of cough frequency monitors.

The cough frequency monitors demonstrated a good ability to discriminate between cough and non‐cough sounds, supporting their accuracy as tools for objective assessment of cough frequency. At present, their restricted use in research environments and lack of investment make expansion into clinical practice technically challenging. Future research should focus on improving the accuracy of monitors, with the goal of enabling clinical availability for patients with cough through the development of devices or mobile apps that are easy to use, cost‐effective, and monitor coughing reliably and in less time.

## Author Contributions

**Ana Lara Castro Rodrigues:** conceptualization, data curation, methodology, resources, validation, visualization, writing – original draft, writing – review and editing, investigation. **Larisse Sousa Reis Passafaro:** conceptualization, investigation, writing – original draft, writing – review and editing, resources, visualization, validation, methodology. **Daniele Oliveira dos Santos:** conceptualization, investigation, writing – original draft, validation, methodology, visualization, writing – review and editing, data curation, resources. **Marcos Gontijo da Silva:** data curation, methodology, software, formal analysis, writing – review and editing, visualization. **Noé Mitterhofer Eiterer Ponce de Leon da Costa:** methodology, visualization, writing – review and editing, software, formal analysis, data curation. **Janne Marques Silveira:** conceptualization, investigation, writing – original draft, methodology, validation, visualization, writing – review and editing, data curation, resources. **Arietta Spinou:** validation, visualization, writing – review and editing. **Ada Clarice Gastaldi:** conceptualization, investigation, writing – original draft, funding acquisition, validation, visualization, writing – review and editing, project administration, data curation, supervision, resources.

## Funding

This work was supported by Coordenação de Aperfeiçoamento de Pessoal de Nível Superior. Fundação de Amparo à Pesquisa do Estado de São Paulo (21/09429‐6, 21/09623‐7, 22/07841‐0). Fundação de Apoio ao Ensino, Pesquisa e Assistência do Hospital das Clínicas da Faculdade de Medicina de Ribeirão Preto da Universidade de São Paulo.

## Ethics Statement

The authors have nothing to report.

## Conflicts of Interest

The authors declare no conflicts of interest.

## Supporting information


**Data S1:** Supporting Information.

## Data Availability

Data used in this study may be obtained upon request to the authors by email at ada@fmrp.usp.br or the Supporting Information.
