# Supplementary material for: Diagnostic Accuracy of Cough Frequency Monitors: A Systematic Review and Meta‐Analysis
Source: Respirology. 2026 Mar 8;31(5):459–70. doi: 10.1002/resp.70229 (PMC13125381; doi:10.1002/resp.70229)
Supplement: Supplementary file 1 — Data S1: Supporting Information. [file RESP-31-459-s001.docx]

**SUPPLEMENTARY MATERIAL**

S1. Concept Map.

| **P** | **I** | **R** | **D** |
| --- | --- | --- | --- |
| “Cough"  [MeSH Terms]  "Cough"  [Title/Abstract]  "Chronic Cough"  [MeSH Terms]  "Chronic Coughs"  [All Fields]  "cough chronic"  [All Fields]  "persistent cough*"  [All Fields]  "cough persistent"  [All Fields]  "nagging cough"  [All Fields] | "monitoring, physiologic"  [MeSH Terms]  "monitoring physiologic"  [Title/Abstract]  "Physiologic Monitoring"  [All Fields]  "Physiological Monitoring"  [All Fields]  "monitoring physiological"  [All Fields]  "Patient Monitoring"  [All Fields]  "monitoring patient"  [All Fields] | No comparator | "Sensitivity and Specificity"  [MeSH Terms]  "Sensitivity and Specificity"  [Title/Abstract]  "Sensitivity"  [All Fields]  "Specificity"  [All Fields] |

# S2. Search Strategy Conducted.

| **Pubmed**  (n=112) | **Web of Science**  (n=244) | **Embase**  (n=10) |
| --- | --- | --- |
| "Cough"[MeSH Terms] OR "Cough"[Title/Abstract] OR "Chronic Cough"[MeSH Terms] OR "Chronic Coughs"[All Fields] OR "cough chronic"[All Fields] OR "persistent cough*"[All Fields] OR "cough persistent"[All Fields] OR "nagging cough*"[All Fields]  **AND**  "monitoring, physiologic"[MeSH Terms] OR "monitoring physiologic"[Title/Abstract] OR "Physiologic Monitoring"[All Fields] OR "Physiological Monitoring"[All Fields] OR "monitoring physiological"[All Fields] OR "Patient Monitoring"[All Fields] OR "monitoring patient"[All Fields]  **AND**  "Sensitivity and Specificity"[MeSH Terms] OR "Sensitivity and Specificity"[Title/Abstract] OR "Sensitivity"[All Fields] OR "Specificity"[All Fields] | **P – Topic**  (Cough OR Chronic Cough) (Topic)  **AND**  **I- Topic**  (Monitoring Patient OR Patient Monitor OR Physiological Monitoring) (Topic)  **AND**  **D- Topic**  (sensitivity AND Specificity OR sensitivity OR Specificity) (Topic) | **P – Title and Abstract**  ('coughing'):ab,ti OR (('chronic cough'):ab,ti)  **AND**  **I – Title and Abstract**   ('coughing'):ab,ti OR (('chronic cough'):ab,ti)  **AND**  **D – Title and Abstract**  ((sensitivity and specificity):ab,ti) |

S3. Software, R packages, versions, and direct URLs used in the analyses.

| **Software / Package** | **Version** | **URL** |
| --- | --- | --- |
| *Mada* | 2024.0.5.12 | [https://CRAN.R-project.org/package=mada](https://cran.r-project.org/package=mada) |
| *RStudio* | 2025.09.2 | [www.posit.co](http://www.posit.co) |
| *R statistical language* | 2025.4.5.2 | [www.r-project.org](http://www.r-project.org) |

# S4. Score of Diagnostic Odds Ratio values, positive likelihood ratio, and negative likelihood ratio.

| **Positive likelihood ratios (LR+)** | |
| --- | --- |
| LR+ >10 | Excellent test for confirming the disease¹ |
| **Negative likelihood ratios (LR-)** | |
| LR- <0,1 | Excellent test to rule out the disease¹ |
| **Diagnostic Odds Ratio (DOR)** | |
| DOR = 1 | No discriminatory capacity² |
| DOR between 1 and 5 | Low accuracy² |
| DOR between 5 and 10 | Moderate accuracy² |
| DOR between 10 and 25 | Good accuracy² |
| DOR > 25 | Very good/Excellent accuracy² |
| DOR tending towards infinity | Perfect test² |

S5. List of excluded studies (n= 60).

| **Author** | **Year** | **Title** | **Reason for exclusion** |
| --- | --- | --- | --- |
| Abdelkhalek, M | 2021 | Investigating the Relationship between Cough Detection and Sampling Frequency for Wearable Devices | Not reference of interest |
| Birring, SS | 2011 | New concepts in the management of chronic cough | Not study design of interest |
| Birring, SS | 2006 | Cough frequency, cough sensitivity and health status in patients with chronic cough | Not test index of interest |
| Brinker, ACD | 2021 | Performance Requirements for Cough Classifiers in Real-World Applications | Not test index of interest |
| Chen, YB | 2024 | Smartwatch-based algorithm for early detection of pulmonary infection: Validation and performance evaluation | Not test index of interest |
| Crooks, MG | 2017 | Continuous Cough Monitoring Using Ambient Sound Recording During Convalescence from a COPD Exacerbation | Not reference of interest |
| Crooks, MG | 2021 | Domiciliary Cough Monitoring for the Prediction of COPD Exacerbations | Not outcomes of interest |
| Crooks, MG | 2015 | Objective Measurement of Cough Frequency During COPD Exacerbation Convalescence | Not outcomes of interest |
| Decalmer, SC | 2007 | Chronic cough: how do cough reflex sensitivity and subjective assessments correlate with objective cough counts during ambulatory monitoring? | Not test index of interest |
| Drugman, T | 2013 | Objective study of sensor relevance for automatic cough detection | Not outcomes of interest |
| Larson, EC | 2011 | Accurate and privacy preserving cough sensing using a low-cost microphone Proceedings of the 13th International Conference on Ubiquitous Computing | Conference abstracts |
| Eni, M | 2022 | Cough detection using a non-contact microphone: A nocturnal cough study | Not test index of interest |
| Faruqi, S | 2011 | Quantifying chronic cough: Objective versus subjective measurements | Not outcomes of interest |
| Gross, V | 2007 | Mobile nocturnal long-term monitoring of wheezing and cough | Not outcomes of interest |
| Hamutcu, R | 2002 | Objective monitoring of cough in children with cystic fibrosis | Not outcomes of interest |
| Hirai, K | 2015 | A new method for objectively evaluating childhood nocturnal cough | Not reference of interest |
| Hirai, K | 2022 | A new method for objectively evaluating nocturnal cough in adults | Not reference of interest |
| Hoyos-Barceló, C | 2020 | Evaluation in a Real Environment of a Trainable Cough Monitoring App for Smartphones | Conference abstracts |
| Kaur, S | 2023 | Development and Validation of a Respiratory-Responsive Vocal Biomarker-Based Tool for Generalizable Detection of Respiratory Impairment: Independent Case-Control Studies in Multiple Respiratory Conditions Including Asthma, Chronic Obstructive Pulmonary Disease, and COVID-19 | Not test index of interest |
| Kelsall, A | 2011 | A Novel Approach to Studying the Relationship Between Subjective and Objective Measures of Cough | Not outcomes of interest |
| Mcguinness, K | 2012 | P159 Validation of the VitaloJAK 24 Hour Ambulatory Cough Monitor | Conference abstracts |
| Klco, P | 2018 | Novel computer algorithm for cough monitoring based on octonions | Not test index of interest |
| Koehler, U | 2014 | LEOSound, an innovative procedure for acoustic long-term monitoring of asthma symptoms (wheezing and coughing) in children and adults | Not available in English |
| Laguarta, J | 2020 | COVID-19 Artificial Intelligence Diagnosis Using Only Cough Recordings | Not test index of interest |
| Leconte, S | 2011 | The objective assessment of cough frequency: accuracy of the LR102 device | Not outcomes of interest |
| Lee, KK | 2013 | A Longitudinal Assessment of Acute Cough | Not reference of interest |
| Liu, JM | 2015 | Cough event classification by pretrained deep neural network | Not test index of interest |
| Martinek, J | 2011 | Distinction of cough from other sounds produced by daily activities in the upper airways | Not test index of interest |
| Matos, S | 2006 | Detection of cough signals in continuous audio recordings using hidden Markov models | Not outcomes of interest |
| McGarvey, LP | 2003 | Psychogenic cough in a schoolboy: evaluation using an ambulatory cough recorder | Not outcomes of interest |
| McGuinness, K | 2008 | The Leicester Cough Monitor: a semi-automated, semi-validated cough detection system? | Not study design of interest |
| Monge-Alvarez, J | 2018 | Audio-cough event detection based on moment theory | Not test index of interest |
| Monge-Alvarez, J | 2019 | Robust Detection of Audio-Cough Events Using Local Hu Moments | Not test index of interest |
| Monge-Alvarez, J | 2019 | A Machine Hearing System for Robust Cough Detection Based on a High-Level Representation of Band-Specific Audio Features | Not test index of interest |
| Orlandic, L | 2023 | A Multimodal Dataset for Automatic Edge-AI Cough Detectio | Not test index of interest |
| Oshi, A | 2021 | Enhanced exploration of chronic cough using Improved Convolutional Neural Networks and remote monitoring harnessing Internet of Things (IoT) | Not test index of interest |
| Paul, IM | 2006 | Evaluation of a new self-contained, ambulatory, objective cough monitor | Not outcomes of interest |
| Pentakota, P | 2023 | Screening COVID-19 by Swaasa AI platform using cough sounds: a cross-sectional study | Not test index of interest |
| Pettinati, MJ | 2021 | Automatic and Robust Identification of Spontaneous Coughs from COVID-19 Patients | Not test index of interest |
| Pramono, RXA | 2019 | Automatic Cough Detection in Acoustic Signal using Spectral Features | Not test index of interest |
| Pramono, RXA | 2019 | Automatic Identification of Cough Events from Acoustic Signals | Not test index of interest |
| Proaño, A | 2016 | Protocol for studying cough frequency in people with pulmonary tuberculosis | Not test index of interest |
| Rahman, T | 2022 | QUCoughScope: An Intelligent Application to Detect COVID-19 Patients Using Cough and Breath Sounds | Not test index of interest |
| Raj, AA | 2007 | Clinical assessment of chronic cough severity | Not study design of interest |
| Rhee, H | 2015 | Evaluating the Validity of an Automated Device for Asthma Monitoring for Adolescents: Correlational Design | Not test index of interest |
| Rietveld, S | 1998 | Diagnostics of spontaneous cough in childhood asthma: results of continuous tracheal sound recording in the homes of children | Not test index of interest |
| Kulkarni, S | 2023 | Self-Supervised Audio Encoder with Contrastive Pretraining for Respiratory Anomaly Detection | Not test index of interest |
| Simou, N | 2021 | A Universal System for Cough Detection in Domestic Acoustic Environments | Not test index of interest |
| Sinha, A | 2016 | Predictors of objective cough frequency in pulmonary sarcoidosis | Not outcomes of interest |
| Smith, J | 2006 | Cough in COPD - Correlation of objective monitoring with cough challenge and subjective assessments | Not outcomes of interest |
| Spinou, A | 2017 | The Objective Assessment of Cough Frequency in Bronchiectasis | Not outcomes of interest |
| Swarnkar, V | 2013 | Neural network based algorithm for automatic identification of cough sounds | Not test index of interest |
| Walter, JR | 2024 | Use of artificial intelligence to develop predictive algorithms of cough and PCR-confirmed COVID-19 infections based on inputs from clinical-grade wearable sensors | Not test index of interest |
| Wang, YX | 2024 | An automatic cough counting method and system construction for portable devices | Not test index of interest |
| Sun, X | 2015 | SymDetector: detecting sound-related respiratory symptoms using smartphones | Conference abstracts |
| Xiao, Y | 2013 | The acoustic cough monitoring and manometric profile of cough and throat clearing | Not test index of interest |
| You, MY | 2022 | Automatic cough detection from realistic audio recordings using C-BiLSTM with boundary regression | Not test index of interest |
| Zhang, J | 2024 | An electronic patient-reported outcome symptom monitor: the Chinese experience with rapid development of a ready-to-go symptom monitor | Not population of interest |
| Zigel, Y | 2016 | Diurnal and seasonal variation of cough episodes in healthy young adults | Not test index of interest |

|  |
| --- |

S6. Assessment of the applicability of the included studies using the QUADAS-2 tool.

A.


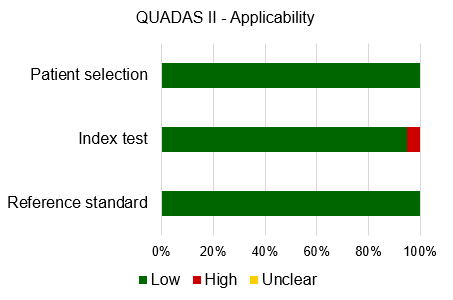


B.


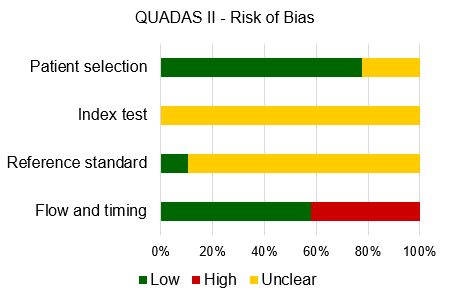


S7. Study data in 2x2 table (TP, FP, FN, TN).

| **Study** | **TP** | **FP** | **TN** | **FN** |
| --- | --- | --- | --- | --- |
| Barata et al., 2023a | 19 | 1 | 54 | 4 |
| Barata et al., 2023b | 11 | 1 | 61 | 5 |
| Barry et al., 2006 | 38 | 8 | 182 | 9 |
| Birring et al., 2008a | 141 | 9 | 868 | 14 |
| Birring et al., 2008b | 95 | 10 | 984 | 15 |
| Coyle et al., 2005 | 284 | 13 | 3268 | 80 |
| Do et al., 2022 | 257 | 0 | 2438 | 14 |
| Hoyos-Barceló et al., 2018 | 4 | 0 | 9 | 0 |
| Kadambi et al., 2018 | 531 | 122 | 4981 | 36 |
| Krajnik et al 2010 | 1452 | 0 | 29433 | 97 |
| Kuhn et al., 2023a | 159 | 0 | 718 | 0 |
| Kuhn et al., 2023b | 67 | 0 | 318 | 13 |
| Kulnik et al., 2016 | 15 | 6 | 56 | 1 |
| Larson et al., 2012 | 113 | 9 | 1335 | 36 |
| Matos et al., 2007 | 16 | 0 | 168 | 3 |
| Otoshi et al., 2021 | 14 | 3 | 84 | 1 |
| Stevens et al., 2024 | 111 | 24 | 584 | 41 |
| Urban et al., 2022 | 129 | 4 | 1296 | 15 |
| Vizel et al., 2010 | 6 | 0 | 6 | 0 |
| TP: true positive; FP: false positive, TN: true negative; FN: false negative. | | | | |

S8. Forest plot of sensitivity and specificity of cough monitors in different subgroups of individuals.


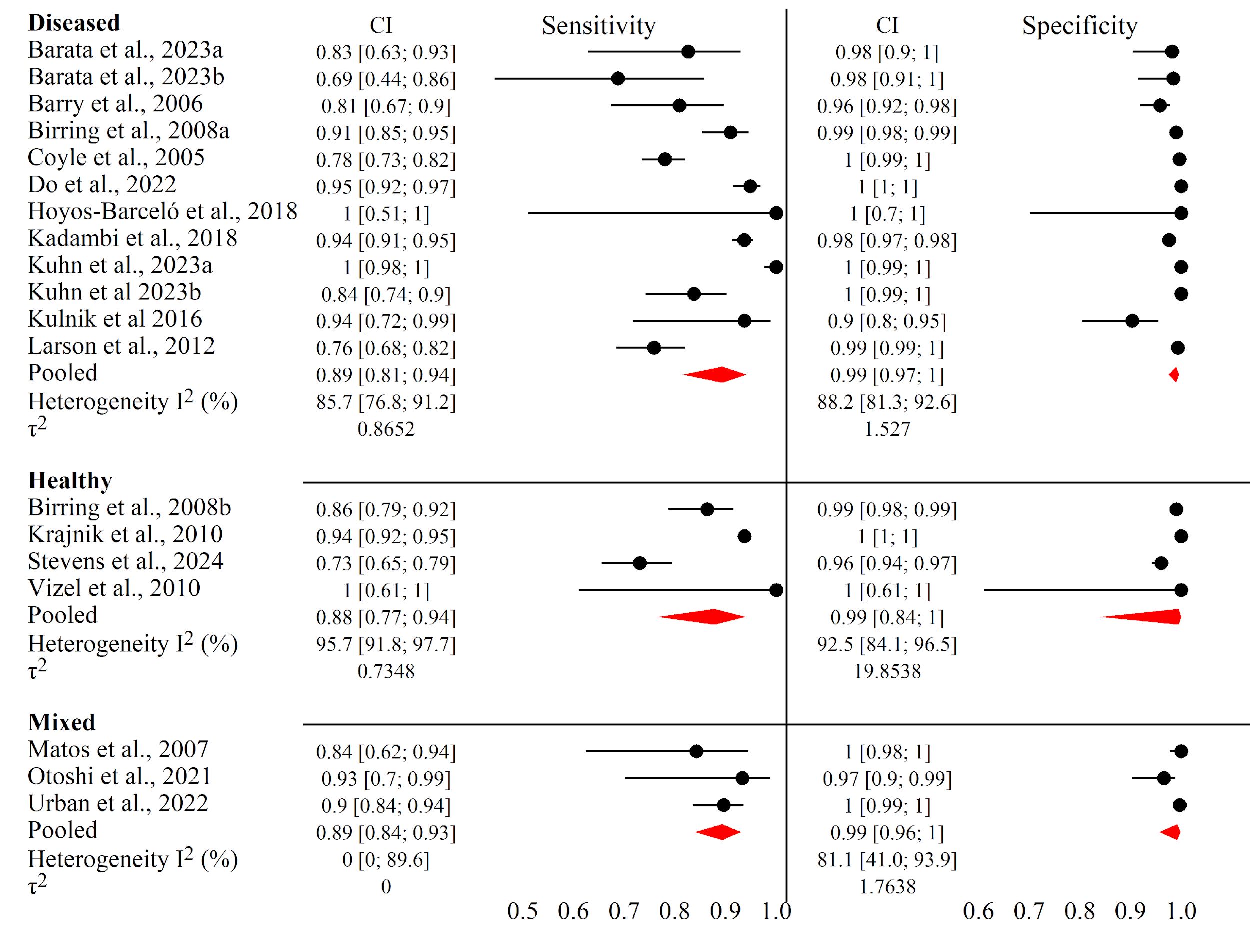


S9. Forest plot of sensitivity and specificity of cough monitors in different locations.


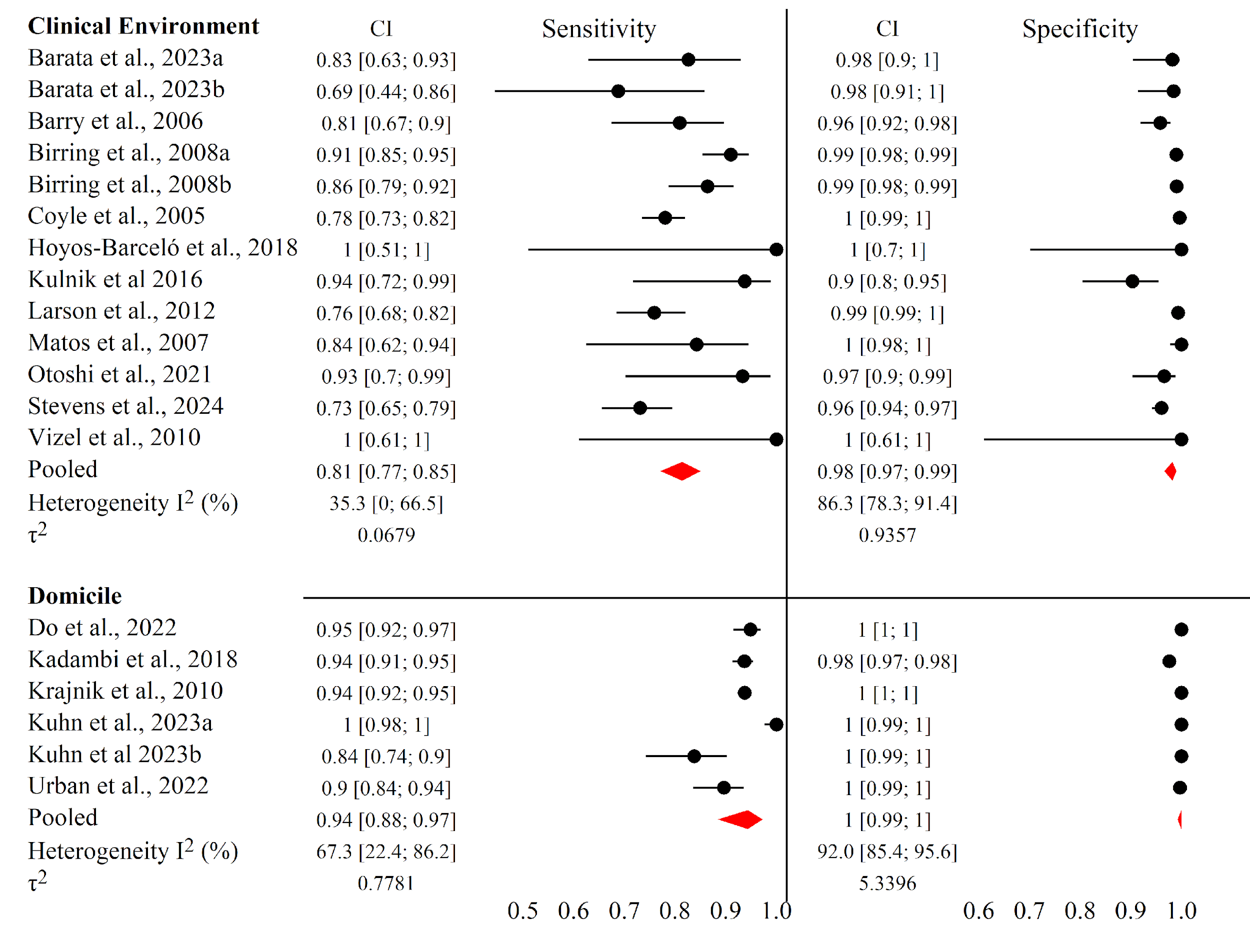


S10. Forest plot of sensitivity and specificity of cough monitors in different subgroups regarding the position of the capture device.


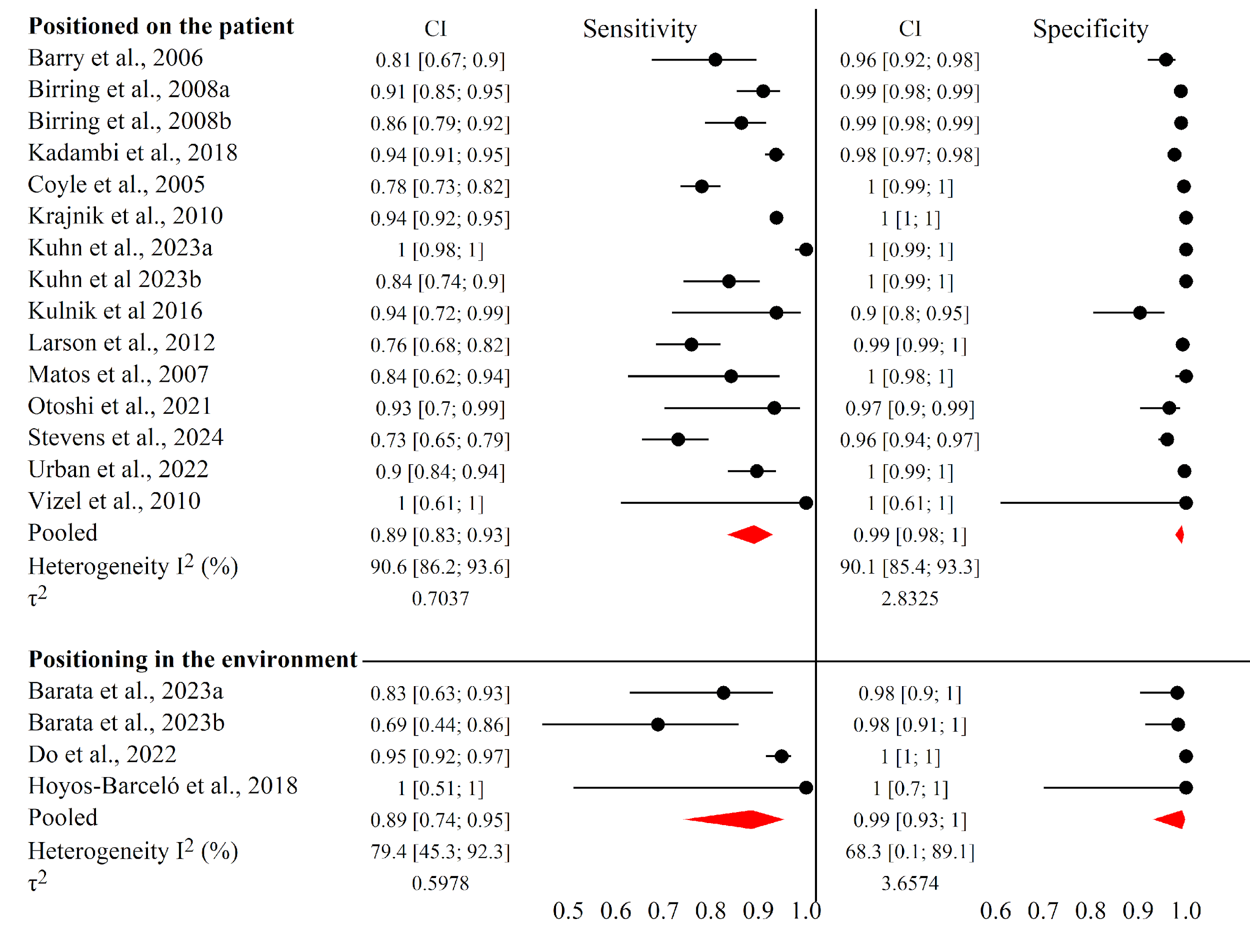


S11. Forest plot of sensitivity and specificity of cough monitors in different patient subgroups according to type of equipment.


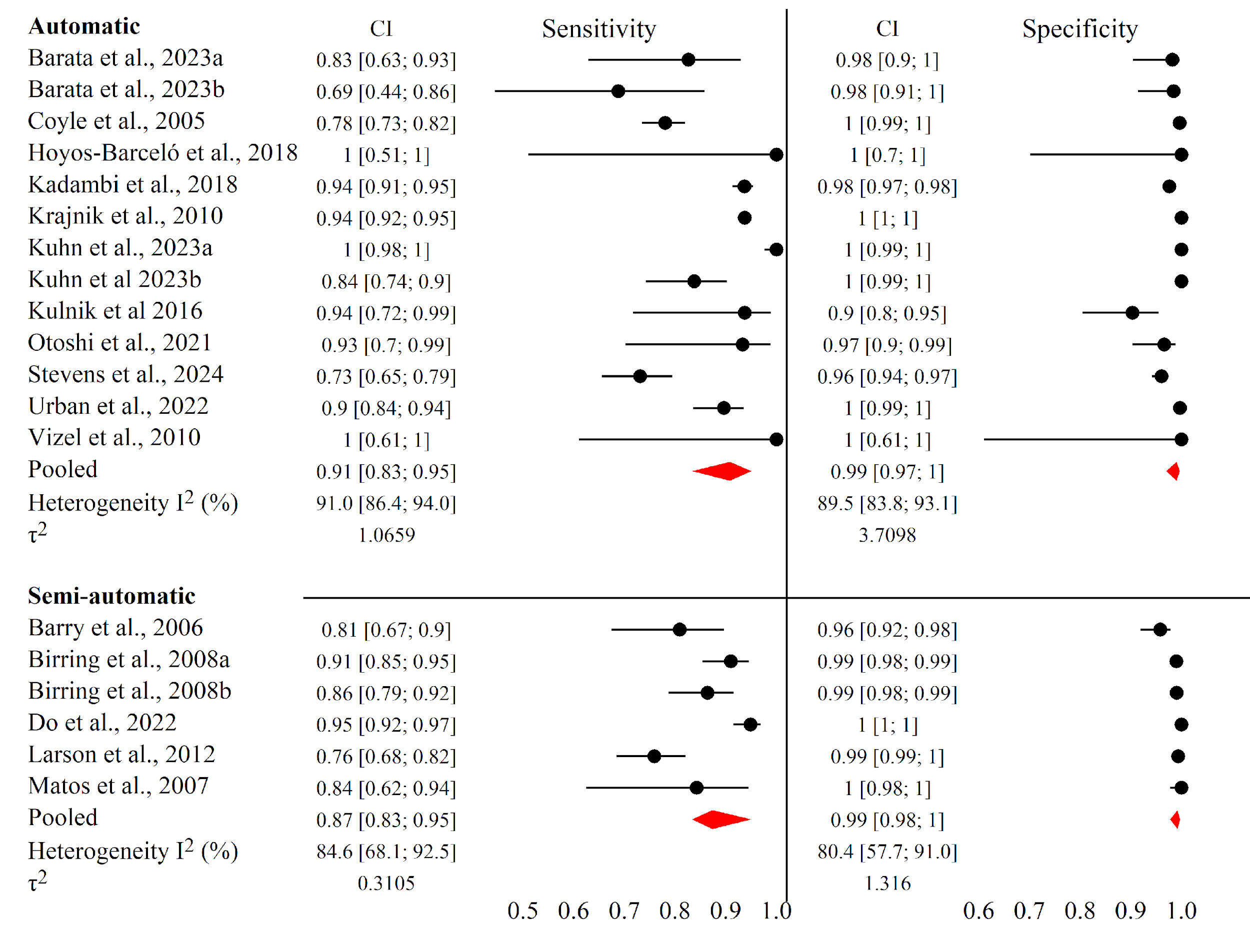


S12. Forest plot of sensitivity and specificity of cough monitors in different patient subgroups according to cough type.


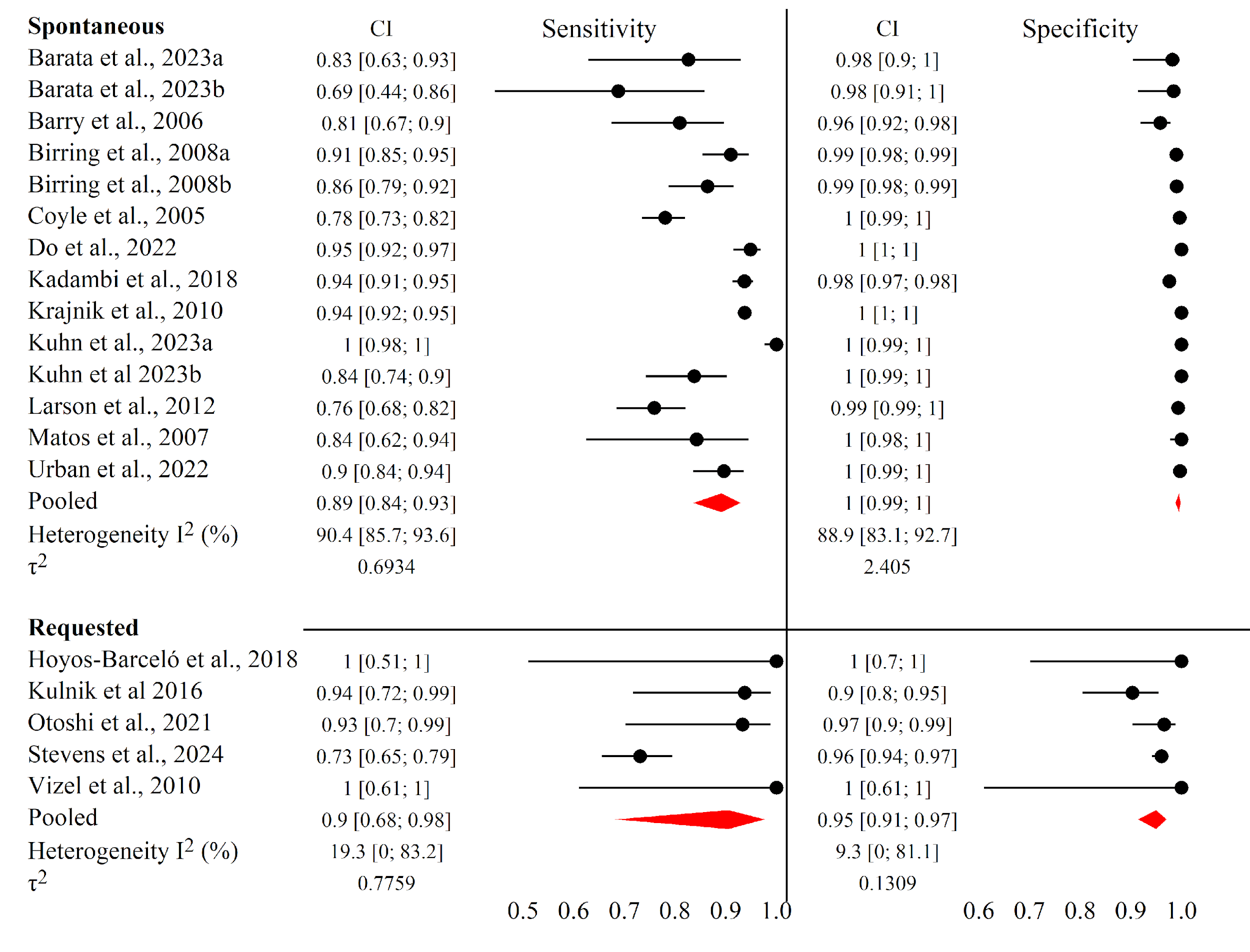


S13. Deeks' primary asymmetry analysis


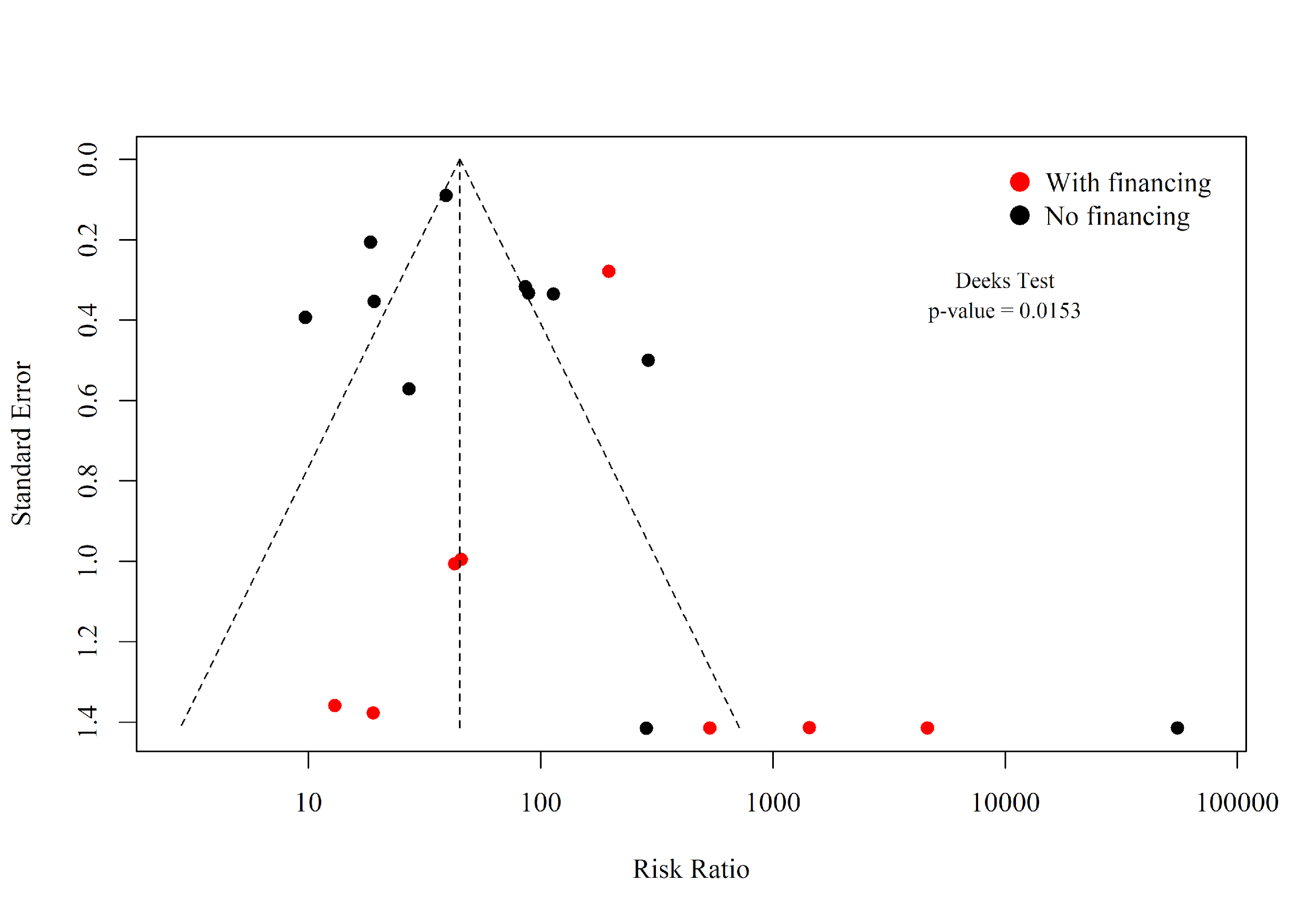


S14. Influence Analysis of Meta-analysis Studies on Diagnostic Accuracy Deviation.

| **Author, year** | **rstudent** | **dffits** | **cook.d** | **tau2.del** | **hat** | **dfbs** | **Influence** |
| --- | --- | --- | --- | --- | --- | --- | --- |
| Barata et al., 2023a | 0.4148 | 0.0761 | 0.0063 | 2.9460 | 0.0646 | 0.0765 |  |
| Barata et al., 2023b | -0.3974 | -0.1216 | 0.0157 | 2.9387 | 0.0486 | -0.1211 |  |
| Barry et al., 2006 | -0.4294 | -0.1278 | 0.0174 | 2.9322 | 0.0483 | -0.1273 |  |
| Birring et al., 2008a | -0.9845 | -0.2648 | 0.0718 | 2.7657 | 0.0635 | -0.2652 |  |
| Birring et al., 2008b | -0.0611 | -0.0571 | 0.0037 | 3.0352 | 0.0638 | -0.0575 |  |
| Coyle et al., 2005 | -0.0799 | -0.0621 | 0.0043 | 3.0366 | 0.0640 | -0.0626 |  |
| Do et al., 2022 | 1.9468 | 0.4551 | 0.1840 | 2.1724 | 0.0381 | 0.4689 |  |
| Hoyos-Barceló et al., 2028 | -0.7674 | -0.1735 | 0.0309 | 2.8246 | 0.0390 | -0.1725 |  |
| Kadambi et al., 2018 | -0.5504 | -0.1783 | 0.0350 | 2.9690 | 0.0662 | -0.1797 |  |
| **Krajnik et al 2010** | **3.5956** | **0.9170** | **0.5981** | **1.3408** | **0.0381** | **1.0243** | ***** |
| Kuhn et al., 2023ª | 1.3003 | 0.2814 | 0.0761 | 2.5163 | 0.0381 | 0.2840 |  |
| Kuhn et al., 2023b | 0.8005 | 0.1551 | 0.0242 | 2.7321 | 0.0381 | 0.1549 |  |
| Kulnik et al., 2016 | -1.4634 | -0.3439 | 0.1079 | 2.4469 | 0.0628 | -0.3420 |  |
| Larson et al., 2012 | 0.0821 | -0.0191 | 0.0004 | 3.0245 | 0.0638 | -0.0193 |  |
| Matos et al., 2007 | 0.4987 | 0.0832 | 0.0071 | 2.8258 | 0.0381 | 0.0827 |  |
| Otoshi et al., 2021 | -0.7386 | -0.2085 | 0.0461 | 2.8805 | 0.0592 | -0.2089 |  |
| Stevens et al., 2024 | -1.0266 | -0.2768 | 0.0778 | 2.7403 | 0.0654 | -0.2771 |  |
| Urban et al., 2022 | 0.6428 | 0.1442 | 0.0218 | 2.8375 | 0.0608 | 0.1445 |  |
| Vizel et al., 2010 | -0.9548 | -0.2046 | 0.0425 | 2.7698 | 0.0394 | -0.2040 |  |

Legend:

- rstudent (Externally Studentized Residual): Measures how well the meta-analysis model fits the effect of each study, considering the exclusion of that study. High absolute values (generally > 2 or 3) indicate a study whose effect deviates considerably from the mean of the other combined effects.
- Dffits (Difference in Fits): A measure of a study's influence on the overall combined effect in the meta-analysis. It quantifies how much the combined effect changes when a specific study is removed.
- cook.d (Cook's Distance): Indicates the impact that removing a study has on the combined result of the meta-analysis.
- tau2.del (Tau-squared Delta): Represents the estimate of tau-squared (τ²) if the study in question is removed from the meta-analysis. It reflects the study's impact on the estimate of heterogeneity among studies.
- hat (Hat Values or Leverage): Measures the distance (or uniqueness) of a study in relation to others in the predictor space. Studies with high "leverage" can have significant influence.
- weight (Weight): The relative weight of the study in the meta-analysis.
- dfbs (dfbeta): Measures the change in the combined effect after the exclusion of a study.
- **Influence: An asterisk (*) in this column indicates that the study is potentially influential, according to the variables combined by the metafor package.**

S15. Deeks’ asymmetry analysis excluding the influential low-prevalence study.
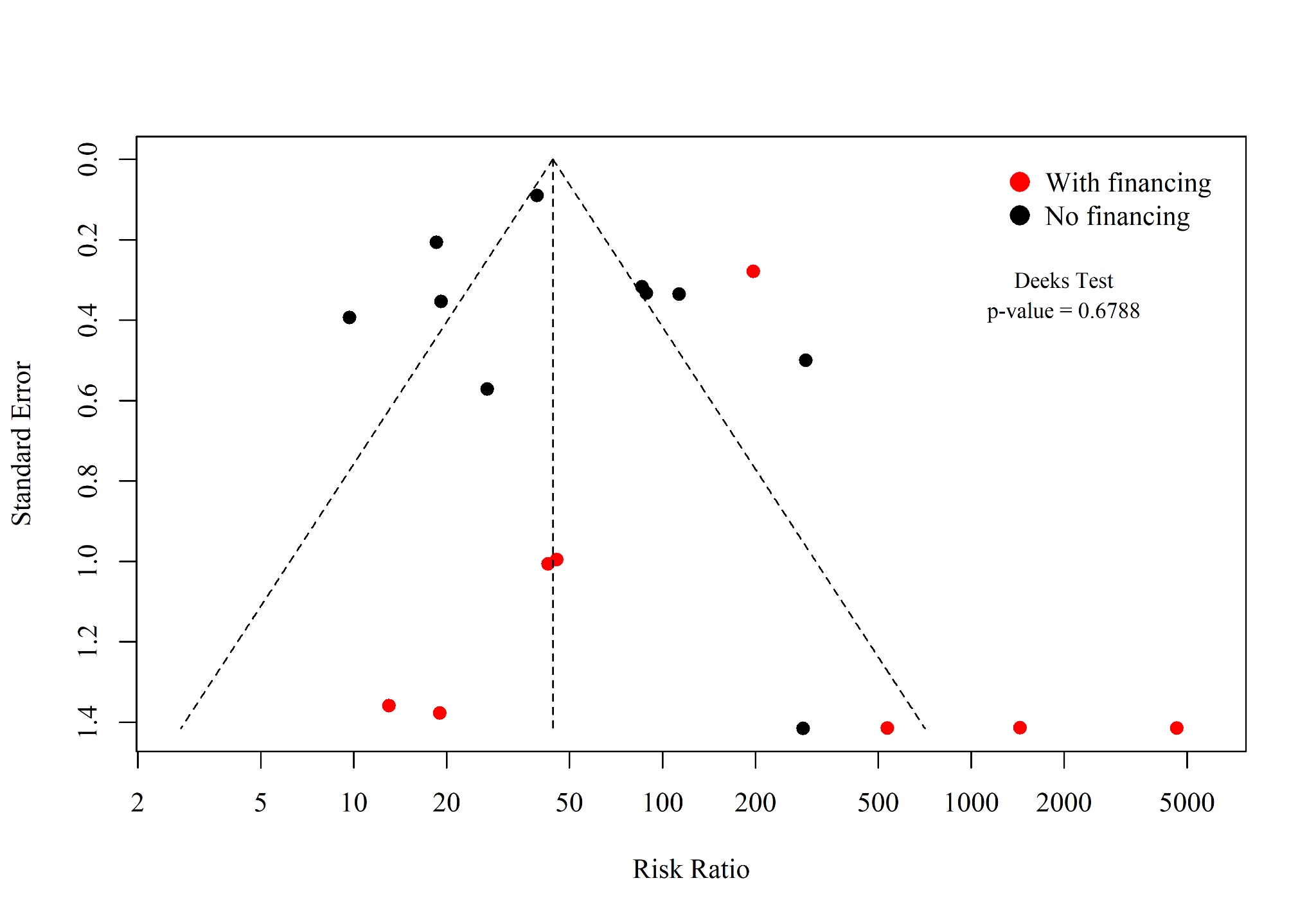


S16. Forest plot of sensitivity and specificity excluding the influential study (Krajnik et al., 2010).


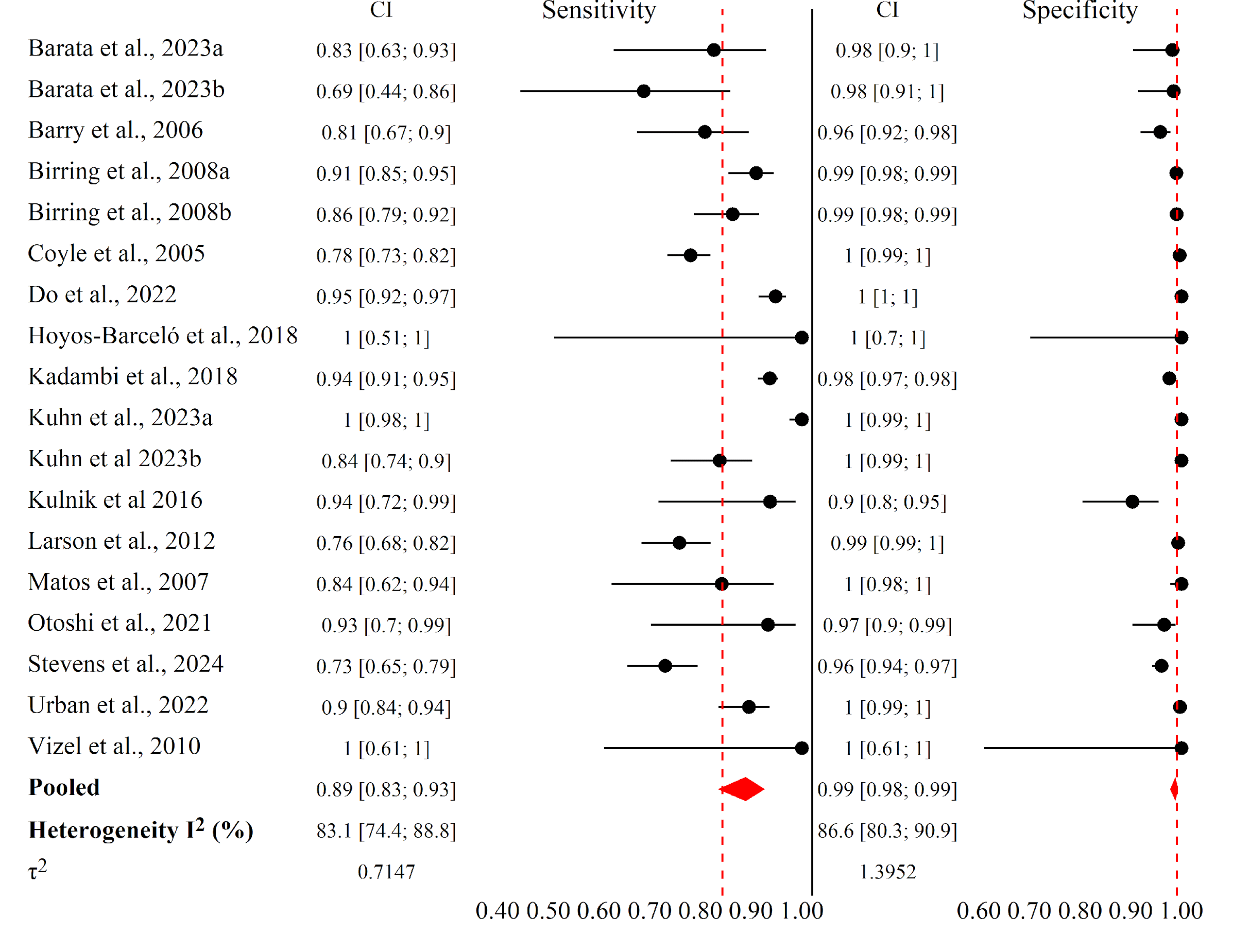


# S17- Certainty of Evidence

| \| **Sensitivity** \| 0.89 (95% CI: 0.93 to 0.84) \|  \|  \| \| --- \| --- \| --- \| --- \| \| **Specificity** \| 0.99 (95% CI: 1.00 to 0.98) \|  \|  \| \| **Positive Likelihood Ratio (LR+)** \| 41.22 (35.00 to 83.56) \|  \|  \| \| **Negative Likelihood Ratio (LR-)** \| 0.09 (0.05 to 0.15) \|  \|  \| \| **Diagnostic Odds Ratio (DOR)** \| 977.34 (333.95 to 4860.35) \|  \|  \| \| **AUC** \| 0.96 (0.93 to 0.97) \|  \|  \| | | | | | | | |  | \| Assumed Prevalence for Predictive Values \| 7.43% \| \| --- \| --- \| | |  |
| --- | --- | --- | --- | --- | --- | --- | --- | --- | --- | --- | --- | --- | --- | --- | --- | --- | --- | --- | --- | --- | --- | --- | --- | --- | --- | --- | --- | --- | --- | --- | --- | --- | --- | --- | --- | --- | --- |
| **Outcome** | **№ of studies (№ of patients)** | **Study design** | **Factors that may decrease certainty of evidence** | | | | | **Positive Predictive Value (PPV) and Negative Predictive Value (NPV)** | | **Test accuracy CoE** |  |
|  |  |  | **Risk of bias** | **Indirectness** | **Inconsistency** | **Imprecision** | **Other considerations** |  |  |  |  |
| **True positives** (patients with cough frequency) | 3395 events  210 events | cross-sectional (cohort type accuracy study) | Serious^a^ | Not serious | Not serious | Not serious | None | PPV: 87.71% | | ⨁⨁⨁◯ Moderada^a^ |  |
| **False negatives** (patients incorrectly classified as not having cough frequency) |  |  |  |  |  |  |  |  |  |  |  |
| **True negatives** (patients without cough frequency) | 46843 events  384 events | cross-sectional (cohort type accuracy study) | Serious^a^ | Not serious | Not serious | Not serious | None | NPV: 99.12% | | ⨁⨁⨁◯ Moderada^a^ |  |
| **False positives** (patients incorrectly classified as having cough frequency) |  |  |  |  |  |  |  |  |  |  |  |

1. The studies did not present the cut-off point, and some did not present blinding.

**Supplementary References**

1. Elkahwagy DMAS, Kiriacos CJ, Mansour M. Logistic regression and other statistical tools in diagnostic biomarker studies. Clin Transl Oncol. 2024;26(9):2172–2180. https://doi.org/10.1007/s12094-024-03413-8
2. Glas AS, Lijmer JG, Prins MH, et al. The diagnostic odds ratio: a single indicator of test performance. Journal of Clinical Epidemiology. 2003;56(11):1129–1135. https://doi.org/10.1016/S0895-4356(03)00177-X
